# Effectiveness of home-based nutritional counselling and support on exclusive breastfeeding in urban poor settings in Nairobi: a cluster randomized controlled trial

**DOI:** 10.1186/s12992-017-0314-9

**Published:** 2017-12-19

**Authors:** Elizabeth W. Kimani-Murage, Paula L. Griffiths, Frederick Murunga Wekesah, Milka Wanjohi, Nelson Muhia, Peter Muriuki, Thaddaeus Egondi, Catherine Kyobutungi, Alex C. Ezeh, Stephen T. McGarvey, Rachel N. Musoke, Shane A. Norris, Nyovani J. Madise

**Affiliations:** 1 African Population and Health Research Center (APHRC), P.O. 10787, Nairobi, 00100 Kenya; 2 International Health Institute, Brown University, Providence, RI USA; 3 College of Medical, Veterinary and Life Sciences, University of Glasgow, Glasgow, UK; 4 Centre for Global Health and Human Development, Loughborough University, Loughborough, UK; 5 MRC/Wits Developmental Pathways for Health Research Unit, Faculty of Health Sciences, University of the Witwatersrand, Johannesburg, South Africa; 6 Julius Center of Health Sciences and Primary Care, Utrecht Medical Center, Utrecht, The Netherlands; 7 Department of Paediatrics, University of Nairobi, Nairobi, Kenya; 8 Centre for Global Health, Population, Poverty, and Policy University of Southampton, Southampton, UK; 9 Stellenbosch Institute for Advanced Study (STIAS), Wallenberg Research Centre at Stellenbosch University, Stellenbosch, South Africa

**Keywords:** Exclusive breastfeeding, Infant feeding practices, Child nutrition, Cluster randomized controlled trials, Kenya, Sub-Saharan Africa, Urban slums

## Abstract

**Background:**

Exclusive breastfeeding (EBF) improves infant health and survival. We tested the effectiveness of a home-based intervention using Community Health Workers (CHWs) on EBF for six months in urban poor settings in Kenya.

**Methods:**

We conducted a cluster-randomized controlled trial in Korogocho and Viwandani slums in Nairobi. We recruited pregnant women and followed them until the infant’s first birthday. Fourteen community clusters were randomized to intervention or control arm. The intervention arm received home-based nutritional counselling during scheduled visits by CHWs trained to provide specific maternal infant and young child nutrition (MIYCN) messages and standard care. The control arm was visited by CHWs who were not trained in MIYCN and they provided standard care (which included aspects of ante-natal and post-natal care, family planning, water, sanitation and hygiene, delivery with skilled attendance, immunization and community nutrition). CHWs in both groups distributed similar information materials on MIYCN. Differences in EBF by intervention status were tested using chi square and logistic regression, employing intention-to-treat analysis.

**Results:**

A total of 1110 mother-child pairs were involved, about half in each arm. At baseline, demographic and socioeconomic factors were similar between the two arms. The rates of EBF for 6 months increased from 2% pre-intervention to 55.2% (95% CI 50.4–59.9) in the intervention group and 54.6% (95% CI 50.0–59.1) in the control group. The adjusted odds of EBF (after adjusting for baseline characteristics) were slightly higher in the intervention arm compared to the control arm but not significantly different: for 0–2 months (OR 1.27, 95% CI 0.55 to 2.96; *p* = 0.550); 0–4 months (OR 1.15; 95% CI 0.54 to 2.42; *p* = 0.696), and 0–6 months (OR 1.11, 95% CI 0.61 to 2.02; *p* = 0.718).

**Conclusions:**

EBF for six months significantly increased in both arms indicating potential effectiveness of using CHWs to provide home-based counselling to mothers. The lack of any difference in EBF rates in the two groups suggests potential contamination of the control arm by information reserved for the intervention arm. Nevertheless, this study indicates a great potential for use of CHWs when they are incentivized and monitored as an effective model of promotion of EBF, particularly in urban poor settings. Given the equivalence of the results in both arms, the study suggests that the basic nutritional training given to CHWs in the basic primary health care training, and/or provision of information materials may be adequate in improving EBF rates in communities. However, further investigations on this may be needed. One contribution of these findings to implementation science is the difficulty in finding an appropriate counterfactual for community-based educational interventions.

**Trial registration:**

ISRCTN ISRCTN83692672. Registered 11 November 2012. Retrospectively registered.

## Background

The global strategy for infant and young child nutrition (IYCN) aims to revitalize efforts to protect, promote and support appropriate infant and young child feeding [1]. Bhutta et al. listed the promotion of breastfeeding and providing supportive strategies as one of the ten evidence-based high impact interventions for improvement of infant and child nutrition and survival [2]. Such strategies may include the Baby Friendly Hospital Initiative (BFHI), a global strategy which promotes breastfeeding in maternity wards around the time of delivery and has been shown to be effective in some settings particularly in the more developed countries [3, 4]. However, in less developed countries, where many deliveries do not occur in health facilities, [5] the effectiveness of the BFHI may be limited.

In Kenya, like in other low and middle income countries (LMICs), poor IYCN practices have been documented widely. To optimize IYCN practices in the country, the Government adapted the WHO/UNICEF global IYCN strategy into a national strategy, [1, 6] actualized through the BFHI. However, most activities have been hospital/clinic based with little extension of breastfeeding counselling and support to the mother in the community after discharge. Furthermore, many deliveries do not occur in health facilities, [7, 8] thereby limiting the impact of BFHI on breastfeeding and other infant feeding practices. Recognizing the need to also reach women at the community level, the Ministry of Health has proposed adoption of the Baby Friendly Community Initiative (BFCI), a global initiative, also developed by WHO and UNICEF, which extends the principles of BFHI at the community level, to complement the BFHI in promotion of optimal breastfeeding and other MIYCN practices (http://bit.ly/2iY7fvV).

The effectiveness of community-based interventions which use CHWs to promote health including optimal breastfeeding practices has been documented, especially among difficult-to-reach predominantly rural populations, but rarely among the urban poor [2, 9–11]. In sub-Saharan Africa (SSA), about 60% of urban residents live in slum settlements, [12] where social and health services are limited, and many women either deliver at home or at sub-standard private health facilities [13]. This means that many of these women may not benefit from the counselling on IYCN offered through the BFHI. In the urban slums of Nairobi, a study conducted in 2007 found that barely 2 % of infants were exclusively breastfed for the first six months. Close to half of children under the age of five in these settings were stunted [14]. The reasons given by mothers for poor breastfeeding and infant feeding practices were: lack of adequate breast milk; poor knowledge; lack of support from health professionals to lactating mothers; food insecurity; and women’s occupations that are incompatible with EBF [15, 16]. These findings reflect both individual and structural factors which can be addressed with targeted counselling and other support.

We designed a cluster randomized controlled trial to test the effectiveness of personalized home-based nutritional counselling by CHWs on MIYCN practices, and consequently on morbidity and nutritional outcomes of infants in two Nairobi slums [17]. The focus of this paper is to determine the effectiveness of this intervention on EBF in the first six months.

## Methods

The study protocol is already published [17]. For this paper we only detail methods relevant to the research question.

### Study setting

The study was carried out in two slums of Nairobi, Kenya (Korogocho and Viwandani) where the African Population and Health Research Center (APHRC) operates the Nairobi Urban Health and Demographic Surveillance System (NUHDSS), covering close to 70,000 residents. The two slums are densely populated with roughly 60,000 inhabitants per square km and are characterized by poor housing, lack of basic infrastructure, violence, insecurity, high unemployment rates and poverty, food insecurity and poor health indicators including poor IYCN practices, high levels of malnutrition and mortality [14, 18–22].

### Study design and randomization

**Fig. 1:**
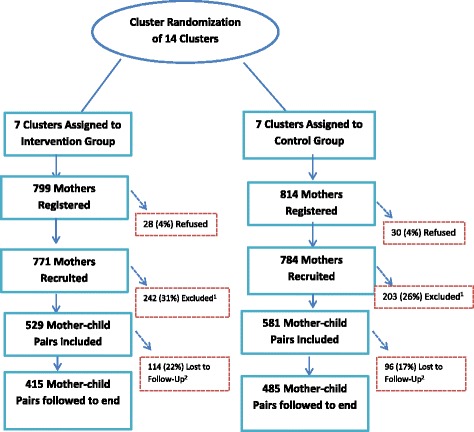
Randomization of Study participants to Intervention and Control Groups, MIYCN Study, Nairobi Slums. ^1^Excluded or dropped due to loss to follow-up during pregnancy due to migration or death of mother, giving birth before receiving the intervention and pregnancy loss (miscarriage/abortion or still birth). ^2^Lost to follow up after giving birth due to migration, or death of mother or the baby

This was a cluster randomized controlled trial [23]. Randomization of the community units (CUs) to the intervention or control arm was computer-generated by a data analyst who was not a primary member of the study team. (A CU as defined by the Kenyan Community Health Strategy is geographically defined with an approximate population of 5000 people. Where the CUs did not exist, APHRC facilitated their set-up). Before randomization, clusters were stratified by slum of residence and the number of women of reproductive age in each cluster (large or small clusters). Fourteen CUs, eight in Korogocho and six in Viwandani were equally randomized into either intervention or control arm. Cluster randomization was preferred over individual-level randomization in order to minimize contamination and for pragmatic purposes as CHWs work in clusters. Figure 1 illustrates the outcome of the randomization process.

### Study subjects

Participants were recruited from any pregnant girls and women aged between 12 and 49 years, who were resident within the defined study area. Girls aged 12–14 years were included because close to 10% of girls below 15 years are sexually active, and from the qualitative work in the study areas young women reported that they need MIYCN counselling [16, 24]. The exclusion criteria were: (a) recruited women who gave birth before receiving the intervention; (b) women with disability that would make delivery of the intervention difficult e.g. intellectual impairment, or who bore a child with a disability that would make feeding difficult; (c) women who lost the pregnancy and/or had a still-birth after being recruited in the intervention; and (d) pregnant women who were lost to follow-up before they delivered.

Efforts to recruit all eligible women were made by using the routine NUHDSS rounds complemented by use of key community informants. All known pregnant women were invited to participate in the study. The target was to recruit the women as early as possible during pregnancy. After obtaining written informed consent, recruitment was done by the data collectors on a rolling basis from September 2012 to February 2014 until the desired sample size was achieved.

### Sample size

The sample size calculation took into account clustering of women in the CUs. A minimum sample size for both intervention and control arms of 196 was estimated to have enough power to detect an increase in EBF for six months from baseline rate of 2% in the study setting [15] to 12%. We used a significance level of 5% and power of 80%. We adjusted for expected intra-cluster correlation (ICC) using a design effect of 3.2 based on an ICC of 0.05, according to previous research in the study area [25]. Allowing for a 20% potential attrition, the sample size of 780 mother-child pairs was estimated. To increase usefulness of the secondary outcomes analysis, we increased the sample size, ending up with a sample size of 1100 at the end of the follow-up.

### Intervention

The experimental intervention involved personalized home-based nutritional counselling of women from the time of recruitment until the baby attained one year. Scheduled visits were: pregnancy - monthly until week 34, then weekly until delivery; mother and baby pairs – weekly in the first month then monthly until12 months. Frequency during the fifth month was biweekly to prepare mothers for complementary feeding. CHWs were given a visiting schedule (Appendix 3) with appropriate key messages at each visit depending on the pregnancy gestational age and age of baby. The expected number of scheduled visits were a total of 7 during pregnancy and 17 after delivery. For each visit the CHW was given a sheet detailing what information to ask for and specific message(s) to give to the mother. Nutritional counselling messages encompassed maternal nutrition, immediate initiation of breastfeeding after birth, breast positioning and attachment, exclusive breastfeeding, frequency and duration of breastfeeding, expressing breast milk, storage, handling and feeding of expressed breast milk and lactation management. It also included age-appropriate complementary feeding. Counselling was also informed by the stages of change model [26]. We did not establish or test the HIV status of participants in this study, but the CHWs in the intervention arm were trained on infant feeding in the context of HIV and were expected to incorporate this in the counselling, without establishing HIV status of the mother. Further, the CHWs were advised to counsel mothers to seek further counselling and support at the health facilities in the event they were HIV positive.

To help in the adaptation of the counselling messages and to inform the design of the intervention, a qualitative study was conducted before the roll-out of the intervention [16, 17]. Additionally, consultations were held with key institutions including the Ministry of Health, UNICEF and other organizations working on MIYCN issues in the community.

Intervention CHWs within the study area recruited from the Community Units in the Community Health Strategy were trained using the Community Infant and Young Child Feeding (IYCF) Counselling Package developed by UNICEF and other partners. This package has been adopted by the Kenya Ministry of Health (http://uni.cf/1QavG2g), based on the WHO IYCF integrated course [27]. Each CHW was given a copy of the counselling cards; brightly colored illustrations that depict key infant and young child feeding concepts. For the intervention CHWs, two follow up training workshops with case discussions were also done. The CHWs were also directly observed intermittently while they counselled women in the households and given feedback.

CHWs in the control arm were not trained on MIYCN but were trained (through the regular government facilitated training) together with the intervention CHWs on standard care, which included ante-natal and post-natal care, family planning, water, sanitation and hygiene, delivery with skilled attendance, immunization and community nutrition. We optimized standard care by ensuring that the intended standard care happened. We therefore facilitating the government to set up Community Units where they did not exist through recruitment of CHWs into the units and offering the CHWs with basic training in order to provide standard counselling. We also provided incentives for CHVs as intended in the Community Health Strategy.

Community health workers in the control arm were expected to visit the mothers according to the standard practice prescribed in the Community Health Strategy, which is defined by need, but generally about once a month per household, and usually more frequent around the time of birth. No specific schedule was given to them.

All recruited pregnant women, whether in the intervention or control arm, received standard care which included counselling from CHWs on primary health care and antenatal and postnatal care and information materials regarding MIYCN.

A total of 30 CHWs across the intervention and control arms were involved in the study. The CHWs in both arms were given a monthly incentive of KES 3500 (approx. USD 35), which is within the government’s approved monthly incentive for CHWs but is rarely implemented. Routine monitoring and supervision of the CHWs was conducted primarily by an Intervention Monitor, and sometimes by other members of the project team, and government officers from the community health strategy. In addition midline and endline qualitative studies involving in-depth interviews and focus group discussion with mothers and CHWs were done among both intervention and control group.

An outline of what was given to intervention vs. control group is given on Table 1. The main differences between the intervention and control arms were that in the intervention arm, the CHWs were given specific training on MIYCN and given counselling cards, while in the control group CHWs were not trained on MIYCN. Also, the in the intervention arm, CHWs were given a specific work schedule to follow up mothers, while no schedule was given to the CHWs in the control group. The CHVs in both intervention and control arms had at least primary level education.

**Table 1:** Intervention vs. Control Group, MIYCN Study, Nairobi Slums

Intervention group	Control group
A) Personalized home-based counselling of mothers on maternal, infant and young child nutrition	A) Not Provided
B) Distribution of MIYCN educational materials (Usual Care)	B) Distribution of MIYCN educational materials (Usual Care)
C) Home-based counselling by CHWs on usual care (e.g. ante-natal care, family planning, delivery with skilled attendance, immunization)	C) Home-based counselling by CHWs on usual care (e.g. ante-natal care, family planning, delivery with skilled attendance, immunization)
D) CHWs specifically trained on MIYCN and given counselling cards. Also given a specific work schedule to follow up the mothers	D) CHWs not specifically trained on MIYCN and no specific work schedule given

### Data collection

Interviewer administered questionnaires were used to collect breastfeeding data and information on control variables as described below.

#### Outcome measure

Data on breastfeeding practices were collected every two months until the infant’s first birthday. We used the WHO definition of EBF as “no other food or drink”, not even water, except breast milk for 6 months of life, but allowing the infant to receive ORS, drops and syrups (vitamins, minerals and medicines) [28]. In terms of measuring this, we used a three day recall to determine if the child had been initiated on other foods. Questions that were asked to establish exclusive breastfeeding included: (i) If the child was given anything other than breast milk in the first three days of life; then at each visit (ii) we asked if the child was given anything other than breast milk in the last three days; (iib) If yes to ii, we asked what the child was given and the age of starting the food/drink; (iii) If no to ii, we asked if the child has ever been given food/drink other than breast milk; (iiib) If yes to iii, we asked what the child was given and the age of starting the food/drink. To determine if the child was exclusively breastfed since birth, we used questions i, ii, and iii. So any mother who reported any deviation from the definition was relegated to a nonexclusive breastfeeding group. To determine at what age the child was given anything other than breast milk, we used questions i, iib, and iiib.

#### Control variables

Control variables collected at baseline and at birth (for example place of delivery) included: household food security assessed using the household food insecurity access scale (HIAS) [29], maternal demographic and socio-economic status; household wealth status; proxy for knowledge on EBF defined by mothers’ knowledge that foods/drinks (other than breast milk) should be introduced at six months, and no pre-lacteal feeds in the first three days of birth; and place of delivery, categorized into two: either at a health facility or other (home or TBA facility). This information is summarized in Table 2.

**Table 2:** Baseline distribution of the study participants by demographic and socioeconomic variables between intervention and control arms, MIYCN Study, Nairobi Slums

	Control		Intervention		Total		
	n	%	n	%	n	%	*p*-value^a^
Mother’s age in years							
14–20	158	27.5	159	30.5	317	29.0	
21–24	177	30.8	164	31.5	341	31.1	
25–29	137	23.9	133	25.5	270	24.7	0.206
30–45	102	17.8	65	12.5	167	15.3	
Mother’s marital status?							
Married	504	87.8	444	85.5	948	86.7	0.546
Not Married	70	12.2	75	14.5	145	13.3	
Mother’s highest education level							
Less than Primary	112	19.7	85	16.8	197	18.4	
Primary School	327	57.6	284	56.2	611	56.9	0.609
Secondary School	129	22.7	136	26.9	265	24.7	
Mother’s religion?							
Christian	525	90.4	480	90.7	1005	90.5	0.944
Muslim/Other	56	9.6	49	9.3	105	9.5	
Mother’s Occupation							
Business/self-employment	65	11.5	66	13.1	131	12.2	
Informal employment	60	10.6	31	6.1	91	8.5	
Formal employment	24	4.2	55	10.9	79	7.4	0.128
Unemployed	418	73.7	353	69.9	771	71.9	
Mother’s ethnicity							
Kikuyu	119	20.5	132	25	251	22.6	
Luhya	92	15.8	73	13.8	165	14.9	
Luo	66	11.4	81	15.3	147	13.2	
Kamba	96	16.5	80	15.1	176	15.9	
Missing	126	21.7	92	17.4	218	19.6	0.831
Other	82	14.1	71	13.4	153	13.8	
Mother’s parity							
Null	212	36.5	223	42.2	435	39.2	
One	178	30.6	165	31.2	343	30.9	0.196
Two+	191	32.9	141	26.7	332	29.9	
Household wealth status							
Lower	147	25.3	122	23.1	269	24.2	
Middle	139	23.9	141	26.7	280	25.2	
Upper	125	21.5	143	27	268	24.1	0.691
Missing	170	29.3	123	23.3	293	26.4	
Household food security status							
Food Secure	153	29.7	129	27.2	282	28.5	
Moderately Food Insecure	186	36.2	216	45.6	402	40.7	0.467
Severely Food Insecure	177	34.1	129	27.2	306	30.8	
Mother Knowledgeable on EBF (at baseline)^b^							
No	217	37.41	201	38.29	418	37.83	0.861
Yes	363	62.59	324	61.71	687	62.17	
Place of delivery^c^							
HF delivery	534	95.87	481	94.31	1015	95.13	0.262
Outside HF delivery	23	4.13	29	5.69	52	4.87	

^a^ *P*-values are based on Chi-square that accounts for clustering

^b^Knowledge that food other than breast milk should be introduced at six months

^c^Place of delivery not collected at baseline but during the follow-up

**Table 3:** Practice of Exclusive Breastfeeding by Intervention Status, MIYCN Study, Nairobi Slums

	Control				Intervention					Total			
	n	%	95% CI		n	%	95% CI		*p*-value^1^	n	%	95% CI	
EBF for 0–2 months													
Yes	419	79.7	76.0	82.9	394	83.5	79.8	86.6		813	81.5	78.9	83.8
No	107	20.3	17.1	24.0	78	16.5	13.4	20.2	0.466	185	18.5	16.2	21.1
EBF for 0–4 months													
Yes	338	69.4	65.2	73.3	307	70.1	65.6	74.2		645	69.7	66.7	72.6
No	149	30.6	26.6	34.8	131	29.9	25.8	34.4	0.929	280	30.3	27.4	33.3
EBF at for 0–6 months													
Yes	250	54.6	50.0	59.1	232	55.2	50.4	59.9		482	54.9	51.6	58.2
No	208	45.4	40.9	50.0	188	44.8	40.1	49.6	0.941	396	45.1	41.8	48.4

^1^ *P*-values are computed after excluding the missing/don’t knows, and after adjusting for clustering

**Table 4:** Logistic regression for exclusive breastfeeding for six months by intervention arm controlling for baseline characteristics, MIYCN Study, Nairobi Slums

	OR(Unadj)	*p*-value	95% CI	OR(Adj)^a^	*p*-value	95% CI
Intervention Group (ref: Control)	1.03	0.941	0.48–2.20	1.11	0.718	0.61–2.02
Child Sex (ref: Male)	0.88	0.127	0.74–1.04	0.80	0.035	0.66–0.98
Mother’s age (Ref: 30–45)						
14–20	0.74	0.096	0.52–1.06	0.99	0.945	0.66–1.48
21–24	0.68	0.024	0.50–0.94	0.81	0.325	0.52–1.27
25–29	0.74	0.109	0.50–1.08	0.78	0.149	0.54–1.11
Mother’s marital status (ref: Married)	0.74	0.158	0.47–1.15	0.60	0.004	0.44–0.82
Mother’s highest completed education level (ref: <Primary)						
Primary School	0.96	0.806	0.66–1.39	0.95	0.768	0.67–1.36
Secondary School	0.86	0.469	0.56–1.33	1.01	0.956	0.63–1.63
Mother’s religion (ref: Christian)	0.95	0.858	0.52–1.74	0.51	0.072	0.25–1.07
Mother’s occupation (ref: Unemployed)						
Business	1.26	0.239	0.84–1.89	0.95	0.798	0.60–1.50
Informal employment	0.91	0.572	0.65–1.28	0.74	0.150	0.49–1.13
Formal employment	0.35	0.001	0.21–0.59	0.31	0.000	0.18–0.54
Mother’s ethnicity (ref: Kikuyu)						
Luhya	0.89	0.583	0.56–1.41	0.85	0.507	0.50–1.44
Luo	0.90	0.595	0.61–1.35	0.82	0.240	0.58–1.16
Kamba	0.80	0.154	0.59–1.10	0.97	0.869	0.66–1.42
Other groups	0.50	0.003	0.33–0.76	0.64	0.151	0.34–1.20
Mother’s parity (ref: Null)						
One	1.15	0.420	0.80–1.64	1.23	0.263	0.84–1.79
Two+	1.44	0.042	1.01–2.05	1.44	0.228	0.77–2.70
Household wealth status (ref:Lowest)						
Middle	0.72	0.390	0.32–1.61	0.69	0.347	0.30–1.57
Upper	0.54	0.105	0.25–1.16	0.52	0.034	0.29–0.94
Household food insecurity status (ref: Food Secure)						
Moderate Food Insecure	1.07	0.744	0.70–1.62	0.92	0.678	0.60–1.41
Severely Food insecure	1.25	0.436	0.69–2.25	0.90	0.658	0.53–1.51
Place of delivery (ref: health facility)						
Outside health facility	0.75	0.381	0.37–1.50	0.58	0.061	0.33–1.03
Mother knowledgeable on EBF (at baseline) (Ref: No)^b^	1.45	0.006	1.13-1.86	1.58	0.002	1.23–2.02

^a^Intracluster Correlation (ICC) of 12.7% was adjusted for

^b^Knowledge that food/drinks other than breast milk should be introduced at six months

#### Statistical analysis

We used the Chi-square test, and adjusted for the cluster study design, baseline differences to compare the proportions of mother-child pairs practicing exclusive breastfeeding (EBF) for two, four and six months The attrition rate was variable between the intervention and control groups (22% versus 17%), and to account for any potential bias from selective attrition we used logistic regression and the baseline characteristics to provide adjusted odds ratios. The cluster study design was taken into account for both the adjusted and unadjusted odds ratios. Intention to treat analysis [30] was applied as appropriate. Among those who were lost to follow-up, last observation carried forward (LOCF) was applied for those whose status as “not EBF” had been determined in the previous rounds of observation. For those whose status was not already established for any time point say two, four or six months (still exclusively breastfeeding in the last observation), LOCF was only used for the point that it was conclusively established, but was not used for latter points. Such an observation was considered as right-censored [31]. Quantitative data analysis was done using Stata version 12.1 (StataCorp LP, College Station, Texas, USA). Statistical significance was assessed with alpha = 0.05 (95% CI).

### Ethical approval

Ethical approval was granted by the Kenya Medical Research Institute (KEMRI) Ethical Review Committee (Reference number: KEMRI/RES/7/3/1). Written informed consent was obtained from all participants. Proxy consent for children was obtained from their mothers.

## Results

As shown in Fig. 1 (CONSORT diagram), a total of 1613 pregnant women were approached for recruitment, 799 in the intervention and 814 in the control clusters. Of these, 58 (4%) refused to be recruited into the study. Of those recruited, 242 (31%) in the intervention group and 203 (26% in the control group) were excluded because they lost the pregnancy, moved away from the study area, died, or because they gave birth before they received the intervention. Therefore, 1110 mother-child pairs were included in the study, 529 in the intervention and 581 in the control group. Of these, 210 (18.9%) were lost to follow-up at different time points in the study after delivery, 114 (22%) in the intervention and 96 (17%) in the control group, but were included in the analysis with respect to the intention to treat principle [30]. The high level of attrition may be attributed to high mobility rates in slum settings, facilitated by search for employment, with 22.5% out-migration annually [21].

### Baseline characteristics

The baseline distribution of the participants by demographic and socioeconomic variables between the intervention and control arms of the study is presented in Table 2. The distributions show no significant difference in basic socio-demographic factors between the two arms. At baseline the majority of mothers (63% in the control and 62% in the intervention arms) knew about the proper timing of introduction of complementary foods. The majority of the children, 94% in the intervention and 96% in the control groups were born at health facilities (see Table 2).

As illustrated in Appendices 1 and 2, there was no difference in the baseline characteristics between the intervention and control arms among those who were excluded from the study due to loss to follow except for one variable (parity). A higher proportion (38%) of women excluded in the intervention group had no children compared to those excluded in the control group (29%), *p* = 0.04 (see Appendix 1). Comparison of characteristics of women who were included and those who were excluded showed that there were no differences except in religion and household food security. A lower proportion of those included was Muslim (9.5%) compared to those who were excluded (13%), while a higher proportion of those included was from food secure households (28%) compared to those excluded (21.5%). *P* < 0.001 (see Appendix 2).

### Exclusive breastfeeding

Table 3 shows the proportions of children that were exclusively breastfed for two, four and six months, measured longitudinally.

A slightly higher proportion of children were breastfed exclusively for at least two months in the intervention group at 83.5% (95% CI 79.8–86.6) compared to the control group at 79.7% (95% CI 76.0–82.9), but the difference was not statistically significant. The prevalence of exclusive breastfeeding reduced with age: EBF for 0–4 months was 70.1% (95% CI 65.6–74.2) for the intervention group and 69.4% (95% CI 65.2–73.3) for the control group, while for 0–6 months this was 55.2% (95% CI 50.4–59.9) in the intervention group and 54.6% (95% CI 50.0–59.1) in the control group. There was no statistically significant difference in the rates of EBF by intervention status at all the points.

### Regression analysis for exclusive breastfeeding

The unadjusted odds of EBF were slightly higher in the intervention arm compared to the control arm but there was no statistically significant difference. At two months (OR 1.29, 95% CI 0.62 to 2.69; *p* = 0.467); four months (OR 1.03; 95% CI 0.48 to 2.25; *p* = 0.929); and six months (OR 1.03, 95% CI 0.48 to 2.20; *p* = 0.941). The adjusted odds of EBF(after adjusting for baseline characteristics) were also slightly higher in the intervention arm compared to the control arm but not significantly different: for two months (OR 1.27, 95% CI 0.55 to 2.96; *p* = 0.550); four months (OR 1.15; 95% CI 0.54 to 2.42; *p* = 0.696), and six months (OR 1.11, 95% CI 0.61 to 2.02; *p* = 0.718). Adjusted odds ratios of EBF by selected characteristics are shown in Table 4.

## Discussion

This cluster randomized controlled trial to determine the effectiveness of personalized home-based counselling by CHWs on exclusive breastfeeding for six months did not find a difference between the intervention and control arms. However, there was a large increase in both groups from a baseline of 2%, [15] to 55% in both arms. The study suggests that the basic nutritional training given to CHWs in the basic primary health care training, and/or provision of information materials may be adequate in improving EBF rates in communities significantly. However, further investigations to conclude on this may be needed.

Using data obtained through a parallel observation study on comparable women who gave birth in the surveillance area, but were not recruited into this study, EBF rates for 6 months were about 3% [32]. This shows that there was no noticeable change in the low EBF rates in these slums for the mother-baby pairs who were not part of this study. The large difference between the study groups and the comparison group in the parallel observation study may be attributed to regular CHWs’ visits for counselling and support and distribution of information materials to the mothers in both intervention and control groups, motivated by incentivizing CHWs to visit mothers in the study setting, and supervising them, hence optimizing the proposed standard primary health care that is hampered by lack of CHW motivation. However, there was some differences in the design of the two studies (intervention study and the observational study) that could result in difference in the outcomes. Though similar questions were asked to the mothers to establish exclusive breastfeeding in the two studies, mothers in the intervention study were recruited during pregnancy and followed up more regularly, while mothers in the observational study were recruited after birth and had fewer follow-up visits, meaning there would be longer recall periods to remember when exclusive breastfeeding ceased.

Given the nature of the intervention, it was not possible to blind the CHWs. Anecdotal evidence from the fieldworkers suggests that once the CHWs in the control group found out that the intervention group CHWs had received extra training and extra counselling materials (i.e. counselling cards), they vowed to work so hard that the women in their arm would perform better. While we did not train CHWs in the control arm on MIYCN, we discovered (through endline evaluation) that the two groups had similar levels of knowledge of MIYCN. For ethical reasons, both groups were given the standard government MIYCN information materials which may have enhanced the knowledge of the mothers and CHWs in both intervention and control groups. We also provided the same monetary incentives to the CHWs in both groups and routine supervision, which could have motivated the CHWs in both groups to visit the mothers, although we did not specifically explore this.

Nationally, there are campaigns to encourage mothers to practice EBF for six months which may be responsible for the improved national EBF rate from 32% in 2008 to 61% reported in the KDHS in 2014 [8]. Another key change that occurred was the adoption of the free maternity care policy in public health facilities (http://bit.ly/1QsLuZ2) since June 2013. This increased health facility deliveries nationally [8]. The free maternity policy could have led to the promotion of breastfeeding through counselling of mothers during antenatal care or delivery at the health facilities by health care workers. However, while free maternity care might have affected initiation rates, sustaining EBF for six months requires dedication and adequate support. Urban poor settings remain unreached due to poor access to health care and social services [16]. We believe that the regular follow up by CHWs in our study made a difference since the rate of EBF among mothers in the slums who were not in the study remained low during the study period, despite the changes nationally [32]. Other studies have found that regular CHW visits to mothers provide support and encouragement which are needed to overcome social barriers to EBF [33]. It is worth noting that our results may not be directly comparable to the results of the KDHS as the KDHS is a cross-sectional study which uses a 24 h recall method, while our study was a longitudinal one and used a more strict definition of EBF, reporting exclusive breastfeeding from birth to six months as described in the methods section.

Our study found a high level of improvement in EBF rates in both the control and intervention arms. Increases in EBF following home-based counselling have also been documented in other studies in Kenya and other LMICs, [11, 34, 35]. In a randomized controlled trial in urban poor settings in Nairobi, Kenya, Ochola et al... (2012) found that EBF in the group that received home-based intensive counselling (seven visits; one prenatally and six post-natally) by trained peers was 23.6% compared to the facility based arm that only received one counselling (pre-natally) (9.2%) and control group that did not receive counselling (5.6%). The home-based intensive counselling group had significantly higher (four times) likelihood of EBF compared to the control group (RR 4.01; 95% CI 2.30, 7.01; *p* = 0.001) [35]. A cluster randomized trial in Dhaka Bangladesh reported a prevalence of EBF at five months of 70% in the intervention compared to 6% in the control group which was over 10-fold increase in EBF [11]. The intervention group in the Dhaka study received counselling visits by trained lay counselors during the last trimester and post-partum until the child was five months, but no visits were reported for the control group. Wangalwa et al’s uncontrolled pre and post study in an agrarian rural setting in Kenya increased EBF from 20% to 52% through CHWs counselling [36]. Our findings are in line with the results of a systematic review in LMICs that found that community-based peer support decreased the risk of discontinuing EBF significantly as compared to control (RR: 0.71; 95% CI: 0.61–0.82), [34] and another systematic review of 20 trials which found that interventions with a post-natal component were effective in improving breastfeeding practices [37]. While studies have also documented a remarkable effect of hospital-based breastfeeding support around the time of birth on EBF, [4, 38] there is a significant drop in continued exclusive breastfeeding shortly after discharge from the hospital in the absence of continued support at the community level [38].

This study contributes to implementation science knowledge, but the lack of appropriate counterfactual in our study is a key limitation in accurately assessing the effect of the intervention. However, the lack of improvement in EBF among women who were not part of our study helps to explain the potential effect of our intervention [32]. Other limitations in this study may include potential bias in reporting of the primary outcome (exclusive breastfeeding), often associated with self-reported outcomes, particularly due to social desirability in the context of an intervention. However, the fact that we asked several questions longitudinally to determine EBF gives us confidence in the estimates. High mobility in the urban slums [21] meant that some of the participants were lost to follow-up. To minimize attrition bias, we used intention-to-treat analysis [30]. We can also not rule out a Hawthorne effect since mothers who were in the control group knew that they were part of the study because of the frequent measurements on the infants that we took at given time points. Finally, the results from our study show an intra-cluster correlation coefficient (ICC) of about12%. In our sample size calculation we assumed an ICC of 5% which is lower than the true value. The implications of this are that with a higher ICC, we would have needed a bigger sample size than we estimated but given that the change in the outcome variable (2% to 55%) found in this study is much higher than we had estimated (2% to 12%) the under-estimation of the ICC is not problematic. This would have been a problem if the change in EBF prevalence had been small. Given the similar improvements in the percentage of infants being breastfed in both groups, it is unlikely that a larger sample would have led to different findings.

## Conclusions

This study indicates potential effectiveness of home-based nutritional counselling by CHWs in improving EBF. The study also suggests that the basic nutritional training given to CHWs in the basic primary health care training, and/or provision of information materials may be adequate in improving EBF rates in communities significantly. This raises the question on the need to have additional MIYCN training for CHWs as well as the intensive scheduled home-based nutritional counselling visits, which may not adequately be answered in this study, and may need further investigation. In line with findings from other studies, [34–38] the study provides evidence of the importance of antenatal, perinatal and post-natal home-based breastfeeding support. The decision by the Kenyan government to scale-up the BFHI and to adopt the BFCI is likely to be effective in promoting EBF in Kenya. While this study contributes to implementation science knowledge, it demonstrates the difficulty of finding an appropriate counterfactual for community-based educational interventions. Nevertheless, this study indicates a great potential for use of CHWs when they are incentivized and monitored as an effective model of promotion of EBF, particularly in urban poor settings [15, 16]. The results of this study are relevant for sub-Saharan African countries which are implementing or likely to implement the BFCI.

## Appendix 1

**Table 5:** Baseline distribution of those excluded by demographic and socioeconomic variables between intervention and control arms, MIYCN Study, Nairobi slums

	Control		Intervention		Total		
	N	%	N	%	N	%	*P*-Value
Mother’s age in years							
14–20	51	25.5	51	21.8	102	23.5	
21–24	63	31.5	76	32.5	139	32	0.833
25–29	58	29	71	30.3	129	29.7	
30–45	28	14	36	15.4	64	14.7	
Mother’s marital status							
Married	165	81.7	187	80.3	352	80.9	
Not married	37	18.3	46	19.7	83	19.1	0.706
Mother’s highest completed education level							
Less than Primary	43	21.4	40	17.4	83	19.3	
Primary School	116	57.7	143	62.2	259	60.1	0.533
Secondary+	42	20.9	47	20.4	89	20.6	
Mother’s religion							
Christian	174	85.7	213	88	387	87	
Muslim/Other	29	14.3	29	12	58	13	0.472
Mother’s occupation							
Business/ self-employment	29	14.4	30	13	59	13.7	
Informal employment	24	11.9	21	9.1	45	10.4	0.648
Formal employment	11	5.5	17	7.4	28	6.5	
Unemployed	137	68.2	162	70.4	299	69.4	
Mother’s ethnicity							
Kikuyu	42	20.7	45	18.6	87	19.6	
Luhya	35	17.2	39	16.1	74	16.6	
Luo	20	9.9	32	13.2	52	11.7	0.677
Kamba	23	11.3	36	14.9	59	13.3	
Other groups	28	13.8	34	14	62	13.9	
Missing	55	27.1	56	23.1	111	24.9	
Mother’s parity							
Null	59	29.1	91	37.6	150	33.7	
One	77	37.9	66	27.3	143	32.1	0.04
Two+	67	33	85	35.1	152	34.2	
Household wealth status							
Lowest	53	26.1	56	23.1	109	24.5	
Middle	47	23.2	50	20.7	97	21.8	0.079
Highest	38	18.7	71	29.3	109	24.5	
Missing	65	32	65	26.9	130	29.2	
Household food security status							
Food secure	35	21.9	38	21.1	73	21.5	
Moderate Food Insecure	58	36.3	86	47.8	144	42.4	0.067
Severely Food insecure	67	41.9	56	31.1	123	36.2	
Mother knowledgeable on EBF at baseline^a^							
No	64	32.7	78	34.7	142	33.7	0.663
Yes	132	67.3	147	65.3	279	66.3	

^a^Knowledge that food/drinks other than breast milk should be introduced at six months

## Appendix 2

**Table 6:** Comparison of baseline characteristics between those included and those excluded, MIYCN Study, Nairobi Slums

	Excluded		Included		Total		
	N	%	N	%	N	%	*P*-Value
Study group							
Control	203	45.6	581	52.3	784	50.4	0.017
Intervention	242	54.4	529	47.7	771	49.6	
Mother’s age							
14–20	102	23.5	297	26.9	399	25.9	
21–24	139	32	355	32.2	494	32.1	0.188
25–29	129	29.7	273	24.7	402	26.1	
30–45	64	14.7	179	16.2	243	15.8	
Mothers marital status							
Married	352	80.9	948	86.7	1266	82.9	0.206
Not married	83	19.1	145	13.3	262	17.1	
Mothers highest completed education level							
Less than Primary	83	19.3	197	18.4	280	18.6	
Primary School	259	60.1	611	56.9	870	57.8	0.247
Secondary+	89	20.6	265	24.7	354	23.5	
Mother’s religion							
Christian	387	87	1005	90.5	1392	89.5	
Muslim/Other	58	13	105	9.5	163	10.5	0.038
Mother’s main source of livelihood							
Business/self-employment	59	13.7	131	12.2	190	12.6	
Informal employment	45	10.4	91	8.5	136	9	0.488
Formal employment	28	6.5	79	7.4	107	7.1	
Unemployed	299	69.4	771	71.9	1070	71.2	
Mother’s ethnicity							
Kikuyu	87	19.6	251	22.6	338	21.7	
Luhya	74	16.6	165	14.9	239	15.4	
Luo	52	11.7	147	13.2	199	12.8	0.142
Kamba	59	13.3	176	15.9	235	15.1	
Other groups	62	13.9	153	13.8	215	13.8	
Missing	111	24.9	218	19.6	329	21.2	
Mother’s parity							
Null	150	33.7	435	39.2	585	37.6	
One	143	32.1	343	30.9	486	31.3	0.104
Two+	152	34.2	332	29.9	484	31.1	
Household wealth status							
Lowest	109	24.5	269	24.2	378	24.3	
Middle	97	21.8	280	25.2	377	24.2	0.477
Highest	109	24.5	268	24.1	377	24.2	
Missing	130	29.2	293	26.4	423	27.2	
Household food security status							
Food secure	73	21.5	280	28.3	353	26.5	
Moderate Food Insecure	144	42.4	403	40.7	547	41.1	0.035
Severely Food insecure	123	36.2	307	31	430	32.3	
Mother knowledgeable on EBF at baseline^a^							
No	142	33.7	368	34.9	510	34.6	
Yes	279	66.3	687	65.1	966	65.4	0.674

^a^Knowledge that food/drinks other than breast milk should be introduced at six months

## Appendix 3

**Table 7:** Visits schedule for CHWs Counselling on Maternal, Infant and Young Child Nutrition

Visit	Gestation in weeks (months)	What to do/check	Messages to be given
PREGNANCY			
1	8–18 wks (2- 4th mo)	Recruitment	• Value of attending ANC for initial evaluation • Ask if attended 1st ANC visit • Start counselling on adequate diet for mother • Value of taking the iron and folate supplements given at the clinic • Value of tetanus vaccine during pregnancy
2	19–22 wks (5th mo)	Remind mother to go for 2nd ANC visit	• Continue counselling on mother’s diet • Value of attending ANC
3	23–27 wks (6th mo)	Ask if attended 2nd ANC visit (24 -28wks)	Start counselling on • Infant feeding • Birth plan • Value of attending ANC • Continue couseling on maternal nutrition
4	28–32 wks (7th mo)	Remind mother to go for 3rd ANC visit (32wks)	• Value of attending ANC • Value of early initiation of breastfeeding • Continue counselling on mother’s diet
5	33–35 wks (8th mo)	Ask if mother attended 3rd ANC visit; Check birth plan	• Value and duration of exclusive breastfeeding • Give messages on child spacing • Continue counselling on mother’s diet • Birth plan
6	36–37 wks (8th -9th mo)	Remind mother to go for 4th ANC visit at 36 wks	Review • early initiation of breastfeeding, • exclusive breastfeeding • Birth plans • How to care for the baby’s cord • Counsel on maternal nutrition during lactation
7	38–40 wks (9th mo)		If not delivered do as in visit 6

All throughout pregnancy encourage mother to eat well and emphasize value of attending ANC and making birth plans. Refer if unwell at any time

**Table 8:** Visits schedule for CHWs Counselling on Maternal, Infant and Young Child Nutrition

Visit	Age of baby	What to do/check	Messages to be given and action
MOTHER AND BABY			
1	2–3 days	How mother and baby are doing is baby breastfeeding well? Did mother get vitamin A supplementation? Did child get polio and BCG vaccination?	• Counsel on exclusive breastfeeding • Positioning and attachment if mother having difficulty • Importance of hygiene for mother and baby • Keep cord clean and dry • Mother’s diet during breastfeeding
2	7 days	Condition of baby and cord. Baby is now fully breastfeeding Mother’s health and condition of breasts	• To continue exclusive breastfeeding • Keep cord clean and dry • Mother’s hygiene and diet • If baby or mother unwell refer for care at health facility
3	14 days		• Give message on expressing breastmilk • Review message on child spacing
4	21 days		
5	1 month	Baby and mother’s health	• How to maintain exclusive breastfeeding • Give mother message on expressing breastmilk • Importance of the 6 week check up for mother and baby • Immunization
6	2 months	Check mother baby book for immunization (Polio, Pentavalent, Pneumococcal at 6, 10 & 14 weeks) and growth monitoring. Has mother started attending a family planning clinic?	• Counsel on how to combine work with exclusive breastfeeding • Show mother how to express and store breastmilk
7	3 months		
8	4 months		
9	5 months		• Start discussing complementary feeding
10	5.5 months	Check immunisation – if no missed doses; is baby growing well?	• Continue counselling on complementary feeding: the foods to give, food hygiene, frequency and amounts in the 6th month • Vitamin A supplementation
11	6 months	Is baby growing well? Baby due for vitamin A supplementation	• Encourage to continue breastfeeding on demand. Start small amounts of complementary feeds 2 times per day
12	7 months	Continue checking baby’s growth and health Remind mother to take baby for measles immunisation (9 months); vitamin A (12 months)	• Continue breastfeeding on demand • Gradually increase amounts and frequency; give a variety to meet baby’s needs for adequate growth
13	8 months		
14	9		
15	10		
16	11		
17	12		

Always refer mother or baby to a health facility for illnesses or poor growth

## Abbreviations

APHRC: African Population and Health Research Center
BFCI: Baby Friendly Community Initiative
BFHI: Baby-friendly Hospital Initiative
CHW: Community Health Worker
CI: Confidence interval
CONSORT: Consolidated Standards of Reporting Trials
CU: Community Unit
EBF: Exclusive breastfeeding
HIAS: Household food insecurity access scale
ICC: Intra-cluster correlation
IYCF: Infant and young child feeding
IYCN: Infant and young child nutrition
KEMRI: Kenya Medical Research Institute
KES: Kenya shilling
LMICs: LOCF, last observation carried forward; Low- and middle-income countries
MIYCN: Maternal, infant and young child nutrition
NUHDSS: Nairobi Urban Health and Demographic System
OR: Odds Ratio
RR: Relative risk
SSA: Sub-Saharan Africa
TBA: Traditional birth attendant
UNICEF: United Nations Children’s Fund
USA: United States of America
USD: United States Dollar
WHO: World Health Organization
